# Supplementary material for: Rac1 activates non-oxidative pentose phosphate pathway to induce chemoresistance of breast cancer
Source: Nat Commun. 2020 Mar 19;11:1456. doi: 10.1038/s41467-020-15308-7 (PMC7081201; doi:10.1038/s41467-020-15308-7)
Supplement: Supplementary file 1 — Supplementary Information [file 41467_2020_15308_MOESM1_ESM.pdf]

Rac1 activates non-oxidative pentose phosphate pathway to induce chemoresistance of breast cancer

Li et al

Supplementary data

These supplementary data contain eight supplementary figures and legends, seven supplementary tables.

Supplementary Figure 1

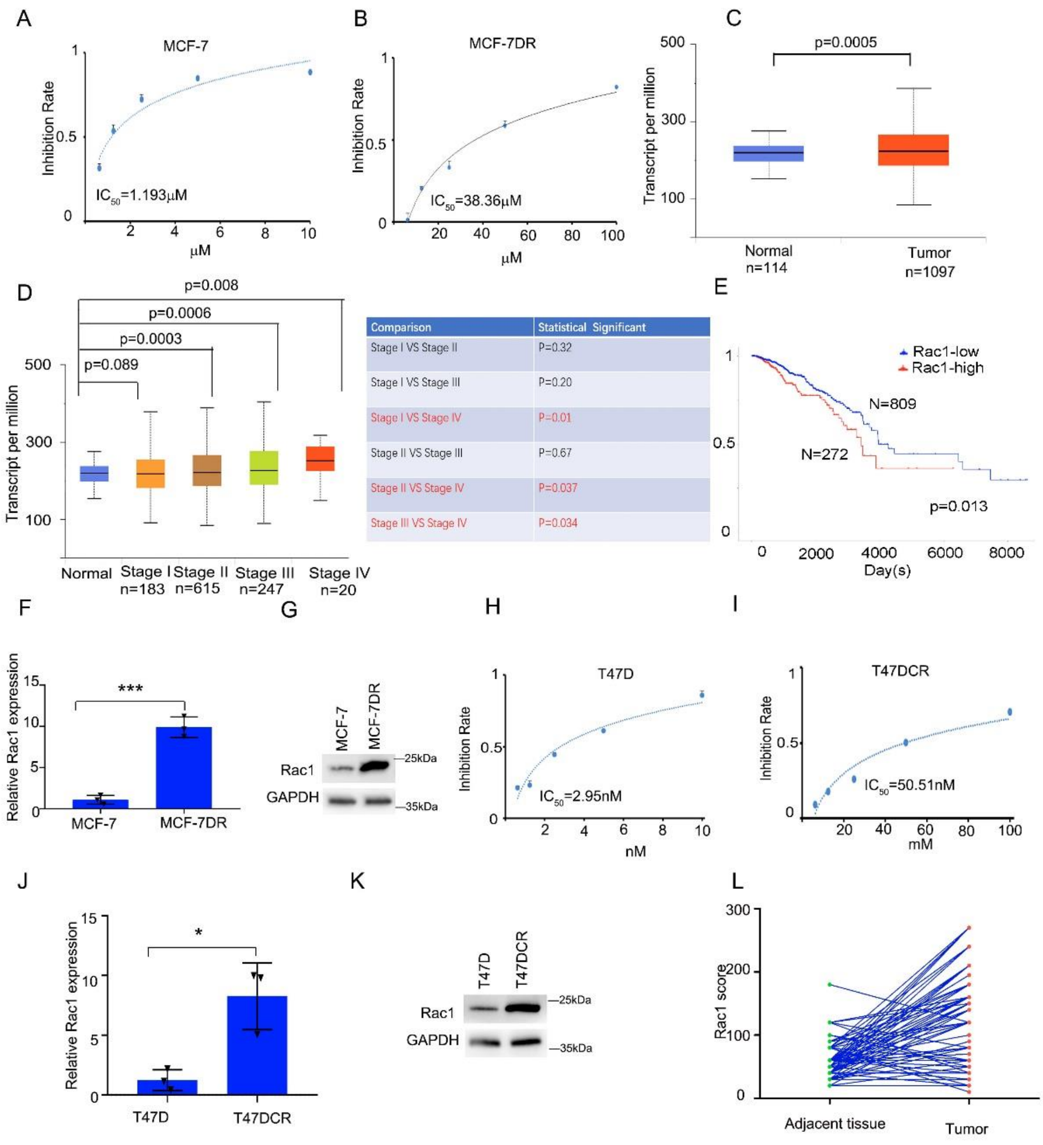

Supplementary Figure 1. Rac1 is upregulated in chemoresistant breast cancer and indicates worse prognosis and neoadjuvant chemotherapy outcome, related to Figure 1

(A-B) IC_{50} of doxorubicin in MCF-7 and MCF-7DR (Doxorubicin-resistant MCF-7). IC50 values were determined according to the experimental triplicates and by fitting the data to Four Parameter Logistic equation using the GraphPad Prism software. Data are presented as mean ± SD.

(C) Rac1 transcript was increased in breast cancer tissues (n=1097) comparing to normal tissues (n=114) in TCGA BRCA database. Center line of box plot represents the median (normal tissue=219.57, tumor =223.22), bounds represent the first (normal tissue=198.43, tumor =187.49) and third quantiles(normal tissue=235.96, tumor =365.86), and whiskers represent the lowest (normal tissue=153.12, tumor =84.29) and highest value (normal tissue=275.58, tumor =287.52, p = 0.0005).The data and graph were obtained from UALCAN website (http://ualcan.path.uab.edu/analysis.html).

(D) Levels of Rac1 transcript in normal and different stages of breast tumors (normal tissue n=144, Stage I n=183, Stage II n=615, Stage III n=247, Stage IV n=20) from TCGA BRCA database. Center line of box plot represents the median (normal tissue=219.569, Stage I =217.811, Stage II =222.804, Stage III =226.477, Stage IV =252.689), bounds represent the first (normal tissue=198.425, Stage I =182.062, Stage II =187.313 Stage III =191.248, Stage IV =225.926) and third quantiles(normal tissue=235.96, Stage I =253.883, Stage II =265.244, Stage III =275.687, Stage IV =286.48), and whiskers represent the lowest (normal tissue=153.12 Stage I =90.428, Stage II =84.289, Stage III =88.381, Stage IV =147.978) and highest value (normal tissue=275.582, Stage I =378.245, Stage II =389.4, Stage III =403.705, Stage IV =317.652). The data and graph were obtained from UALCAN website (http://ualcan.path.uab.edu/analysis.html).

(E) Kaplan-Meier survival curve of breast cancer patients with low and high Rac1-expressing tumors from TCGA BRCA database. The p = 0.013. The data and graph were obtained from UALCAN website (http://ualcan.path.uab.edu/analysis.html).

(F) Real-time PCR for relative mRNA levels of Rac1 in MCF-7and MCF-7DR cells.

(G) Immunoblotting for Rac1 expression in MCF-7 cells and MCF-7DR cells. The result was obtained over three independent experiments. Bar graphs represent the mean ± SD of three independent experiments. (p = 0.0004, two-sided unpaired t-test). *p<0.05, **p < 0.01 and ***p < 0.001.

(H, I) IC_{50} of carboplatin in T47D and T47DCR (carboplatin-resistant T47D). IC50 values were determined according to the experimental triplicates and by fitting the data to Four Parameter Logistic equation using the GraphPad Prism software. Data are presented as mean ± SD.

(J) Real-time PCR for relative mRNA levels of Rac1 in T47D and T47DCR cells. Bar graphs represent the mean ± SD of three independent experiments. (p = 0.0141, two-sided unpaired t-test). *p<0.05, **p < 0.01 and ***p < 0.001.

(K) Immunoblotting for Rac1 expression in T47D and T47DCR cells. The result was obtained over three independent experiments.

(L) Immunohistochemistry score of Rac1 in paraffin-embedded sections of paired breast cancer and adjacent normal tissues from 86 patients. Source data are provided as a Source Data file.

Supplementary Figure 2

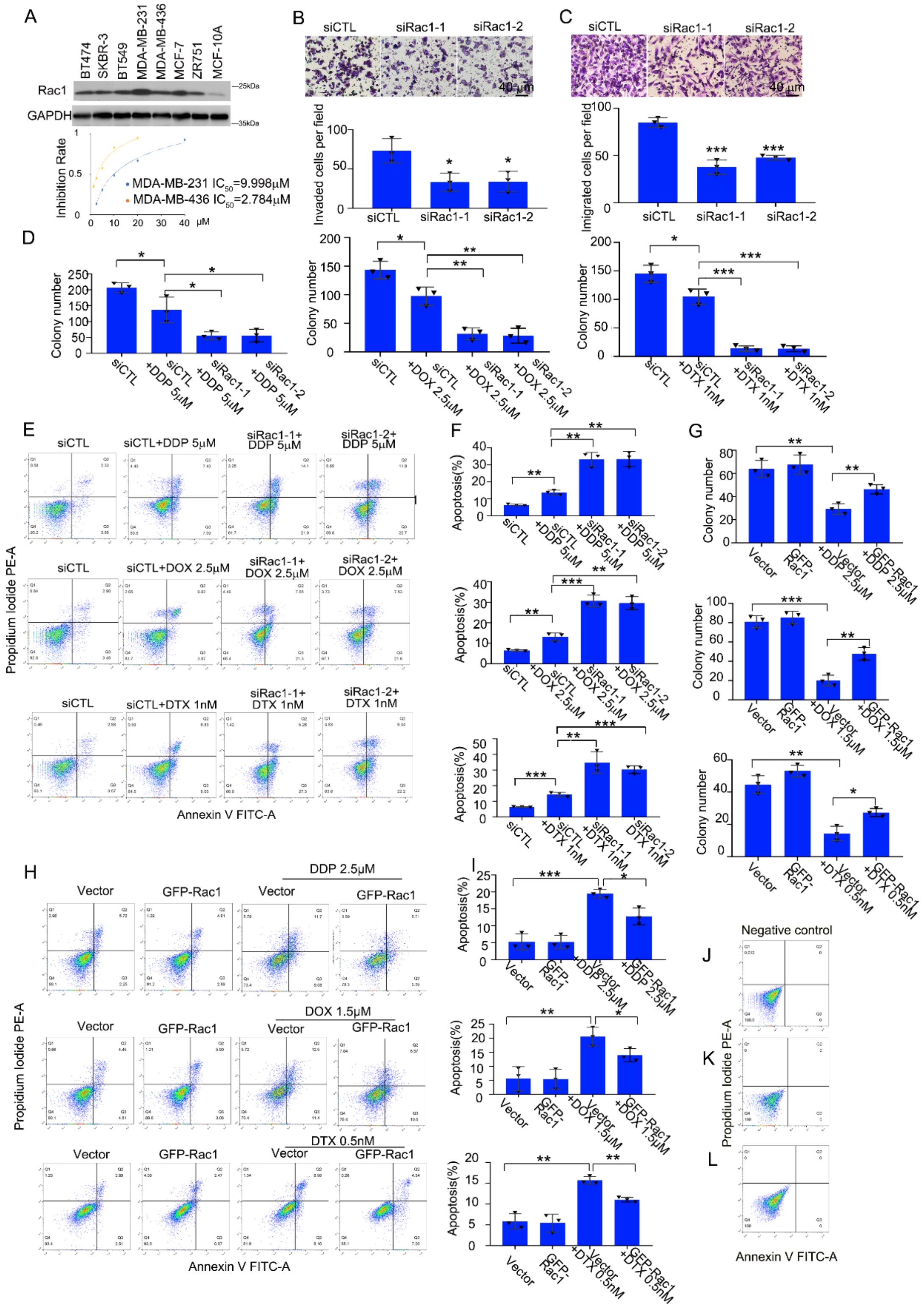

Supplementary figure 2. Rac1 knockdown increases chemosensitivity of breast cancer cell *in vitro*, related to Figure 2

(A) Rac1 levels and IC_{50} of DDP among the mammary cell lines. Rac1 expression in breast cancer cell lines and immortalized mammary epithelial cell line MCF-10A were determined by immunoblotting (upper panel). The result was obtained over three independent experiments. IC_{50} of DDP in MDA-MB-231 and MDA-MB-436 cells (down panel). IC50 values were determined according to the experimental triplicates and by fitting the data to Four Parameter Logistic equation using the GraphPad Prism software.

(B, C) Effects of Rac1 knockdown on cell invasion and migration. Culture inserts were coated with (B) or without (C) matrigel for the Boyden chamber assay. All images were obtained at 100x magnification under an inverted microscope. The result was obtained over three independent experiments. (DDP siCTL vs siCTL+DDP p = 0.0485, siCTL+DDP vs si-1+DDP p = 0.0165, siCTL+DDP vs si-2+DDP p = 0.0364, DOX siCTL vs siCTL+ DOX p = 0.0225, siCTL +DOXvs si-1+ DOX p = 0.0037, siCTL +DOX vs si-2+ DOX p = 0.0042, DTX siCTL vs siCTL+ DTX p = 0.0257, siCTL +DTX vs si-1+ DTX p = 0.0004, siCTL+DTX vs si-2+ DTX p = 0.0004, two-sided unpaired t-test). *p<0.05, **p < 0.01 and ***p < 0.001.

(D) Quantification of colony formation assay of siRac1 MDA-MB-231 cell treated with 5 μM DDP, 2.5 μM doxorubicin or 1nM docetaxel. Bar graphs represent the mean ± SD of three independent experiments. (DDP siCTL vs siCTL+DDP p = 0.0485, siCTL+DDP vs si-1+DDP p = 0.0165, siCTL+DDP vs si-2+DDP p = 0.0364, DOX siCTL vs siCTL+ DOX p = 0.0225, siCTL +DOXvs si-1+ DOX p = 0.0037, siCTL +DOX vs si-2+ DOX p = 0.0042, DTX siCTL vs siCTL+ DTX p = 0.0257, siCTL +DTX vs si-1+ DTX p = 0.0004, siCTL+DTX vs si-2+ DTX p = 0.0004, two-sided unpaired t-test). *p<0.05, **p < 0.01 and ***p < 0.001.

(E, F) Rac1 knockdown increases the chemo drug induced cell apoptosis. SiRac1 MDA-MB-231 cell were treated with 5μM DDP, 2.5μM doxorubicin, or 1nM docetaxel. (DDP siCTL vs siCTL+DDP p = 0.001, siCTL+DDP vs si-1+DDP p = 0.0015, siCTL+DDP vs si-2+DDP p = 0.0018, DOX siCTL vs siCTL+ DOX p = 0.0040, siCTL

+DOXvs si-1+ DOX $p = 0.0009$, siCTL +DOX vs si-2+ DOX $p = 0.0015$, DTX siCTL vs siCTL+ DTX $p = 0.0007$, siCTL +DTX vs si-1+ DTX $p = 0.007$, siCTL+DTX vs si-2+ DTX $p = 0.0007$, two-sided unpaired t-test). Bar graphs represent the mean ± SD of three independent experiments. *$p<0.05$, **$p < 0.01$ and ***$p < 0.001$.

(G) Quantification of colony formation assay of Rac1 overexpressing-MDA-MB-436 cell treated with 2.5μM DDP, 1.5μM doxorubicin, 0.5nM docetaxel. (DDP Vector vs Vector +DDP $p = 0.0021$, Vector +DDP vs GFP-Rac1+DDP $p = 0.0083$, DOX Vector vs Vector + DOX $p = 0.0002$, Vecto +DOX vs GFP-Rac1+ DOX $p = 0.005$, DTX Vector vs Vector + DTX $p = 0.0018$, Vecto +DTX vs GFP-Rac1+ DTX $p = 0.0121$, two-sided unpaired t-test). Bar graphs represent the mean ± SD of three independent experiments. *$p<0.05$, **$p < 0.01$ and ***$p < 0.001$.

(H, I) Rac1 overexpression decreases the chemo drug induced cell apoptosis. Rac1 overexpression MDA-MB-436 cell were treated with 2.5μM DDP, 1.5μM doxorubicin, or 0.5μM docetaxel. (DDP Vector vs Vector +DDP $p = 0.0007$, Vector +DDP vs GFP-Rac1+DDP $p = 0.0143$, DOX Vector vs Vector + DOX $p = 0.0088$, Vector +DOX vs GFP-Rac1+ DOX $p = 0.0476$, DTX Vector vs Vector + DTX $p = 0.0011$, Vector +DTX vs GFP-Rac1+ DTX $p = 0.0017$, two-sided unpaired t-test). Bar graphs represent the mean ± SD of three independent experiments. *$p<0.05$, **$p < 0.01$ and ***$p < 0.001$.

(J-I) In the apoptosis analysis, gating of the flow cytometry data was according to the cells in siCTL or Vector group without Annexin V and Propidium Iodide staining. The gating of Figure 5G and Supplementary Figure 2E were indicated in (J), the gating of DDP and DOX groups in Supplementary Figure 2H were indicated in (K) and the gating of DTX group in Supplementary Figure 2H was indicated in (I).

Source data are provided as a Source Data file.

Supplementary Figure 3

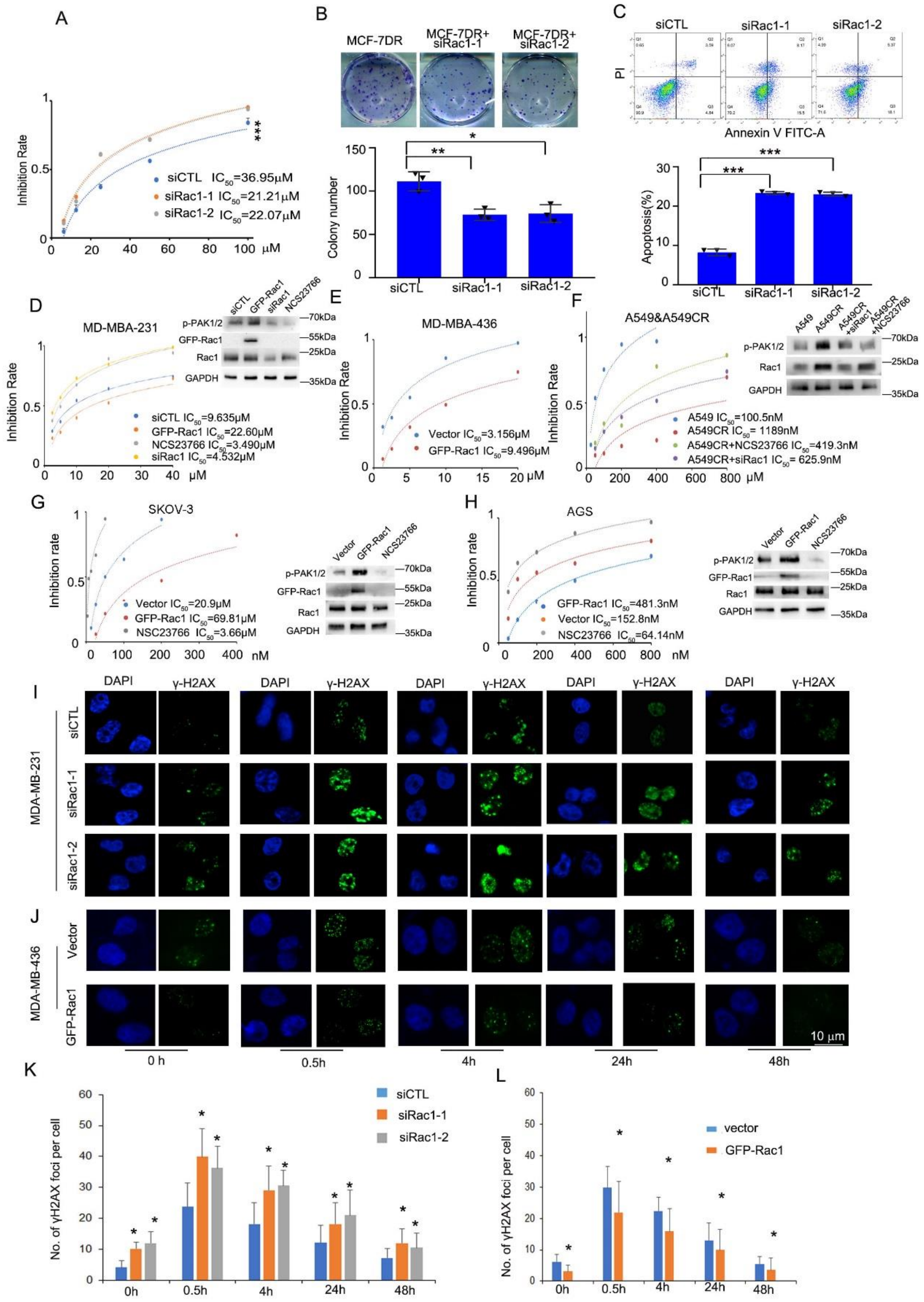

Supplementary figure 3. Rac1 silencing induces DNA damage of breast cancer cells, related to Figure 2

(A) IC_{50} of DOX in MCF-7DR cells transiently transfected with siCTL, siRac1-1 and siRac1-2. (siCTL vs si-1 $p < 0.0001$, siCTL vs sI-2+DDP $p < 0.0001$, two-way ANOVA + Dunnett's post hoc tests). IC50 values were determined according to the experimental triplicates and by fitting the data to Four Parameter Logistic equation using the GraphPad Prism software. *p<0.05, **p < 0.01 and ***p < 0.001.

(B) Rac1 knockdown decreases colony formation of MCF-7DR cells. MCF-7DR cells were treated with doxorubicin (20 μM) after transiently transfected with siCTL, siRac1-1 and siRac1-2 for 48h and then cultured for colony formation. (siCTL vs si-1 p = 0.0062, siCTL vs si-2 p = 0.0124, two-sided unpaired t-test). Bar graphs represent the mean ± SD of three independent experiments. *p<0.05, **p < 0.01 and ***p < 0.001.

(C) Rac1 knockdown induces MCF-7DR apoptosis. MCF-7DR cells were treated with doxorubicin (20 μM) after transiently transfected with siCTL, siRac1-1 and siRac1-2 for 48h and then collected for flowcytometry assay. (siCTL vs si-1 p < 0.0001, siCTL vs si-2 p < 0.0001, two-sided unpaired t-test). Bar graphs represent the mean ± SD of three independent experiments. Bar graphs represent the mean ± SD of three independent experiments. *p<0.05, **p < 0.01 and ***p < 0.001.

(D) IC_{50} of DDP in MDA-MB-231 cells transiently transfected with GFP-Rac1, siCTL, siRac1 or Rac1 inhibitor NCS23766 (50 μM). The immunoblotting was obtained over three independent experiments. IC50 values were determined according to the experimental triplicates and by fitting the data to Four Parameter Logistic equation using the GraphPad Prism software.

(E) IC_{50} of DDP in MDA-MB-436 cells transiently transfected with control vector or GFP-Rac1. IC50 values were determined according to the experimental triplicates and by fitting the data to Four Parameter Logistic equation using the GraphPad Prism software.

(F) IC_{50} of carboplatin in lung adenocarcinoma A549 cells and A549CR (carboplatin - resistant) cells treated with siRac1 or Rac1 inhibitor NCS23766 (50 μM). The immunoblotting was obtained over three independent experiments. IC50 values were

determined according to the experimental triplicates and by fitting the data to Four Parameter Logistic equation using the GraphPad Prism software.

(G-H) IC_{50} of DDP in Skov-3 and AGS cells transiently transfected with control vector, GFP-Rac1 or Rac1 inhibitor NCS23766 (50μM). The immunoblotting was obtained over three independent experiments. IC50 values were determined according to the experimental triplicates and by fitting the data to Four Parameter Logistic equation using the GraphPad Prism software.

(I-L) Rac1 knockdown delays the DNA damage repairing, while Rac1 overexpression accelerates the DNA damage repairing as shown by γH2AX foci formation. Rac1-silencing MDA-MA-231 (I, K) or Rac1 overexpressing MDA-MA-436 (J, L) cells were either treated with or without IR (2 Gy) and allowed to recover for 0.5, 4, 24, or 48h before fixation and processed for γH2AX immunofluorescence. (Figure K n=15, 0h siCTL vs si-1 $p < 0.0001$, siCTL vs si-2 $p < 0.0001$, 0.5h siCTL vs si-1 $p < 0.0001$, siCTL vs si-2 $p = 0.0001$, 4h siCTL vs si-1 $p = 0.0005$, siCTL vs si-2 $p < 0.000$, 24h siCTL vs si-1 $p = 0.0148$, siCTL vs si-2 $p = 0.0019$, 48h siCTL vs si-1 $p = 0.0044$, siCTL vs si-2 $p = 0.0458$, Figure I n=15, 0h Vector vs GFP-Rac1 $p < 0.0001$, 0.5h Vector vs GFP-Rac1 $p = 0.0003$, 4h Vector vs GFP-Rac1 $p = 0.0003$, 24h Vector vs GFP-Rac1 $p = 0.0177$, 48h Vector vs GFP-Rac1 $p = 0.0003$ two-sided unpaired t-test).The scale bar represents 10μm. Bar graphs represent the mean ± SD from 15 independent cells of three independent experiments. *$p<0.05$, **$p < 0.01$ and ***$p < 0.001$.

Source data are provided as a Source Data file.

Supplementary Figure 4

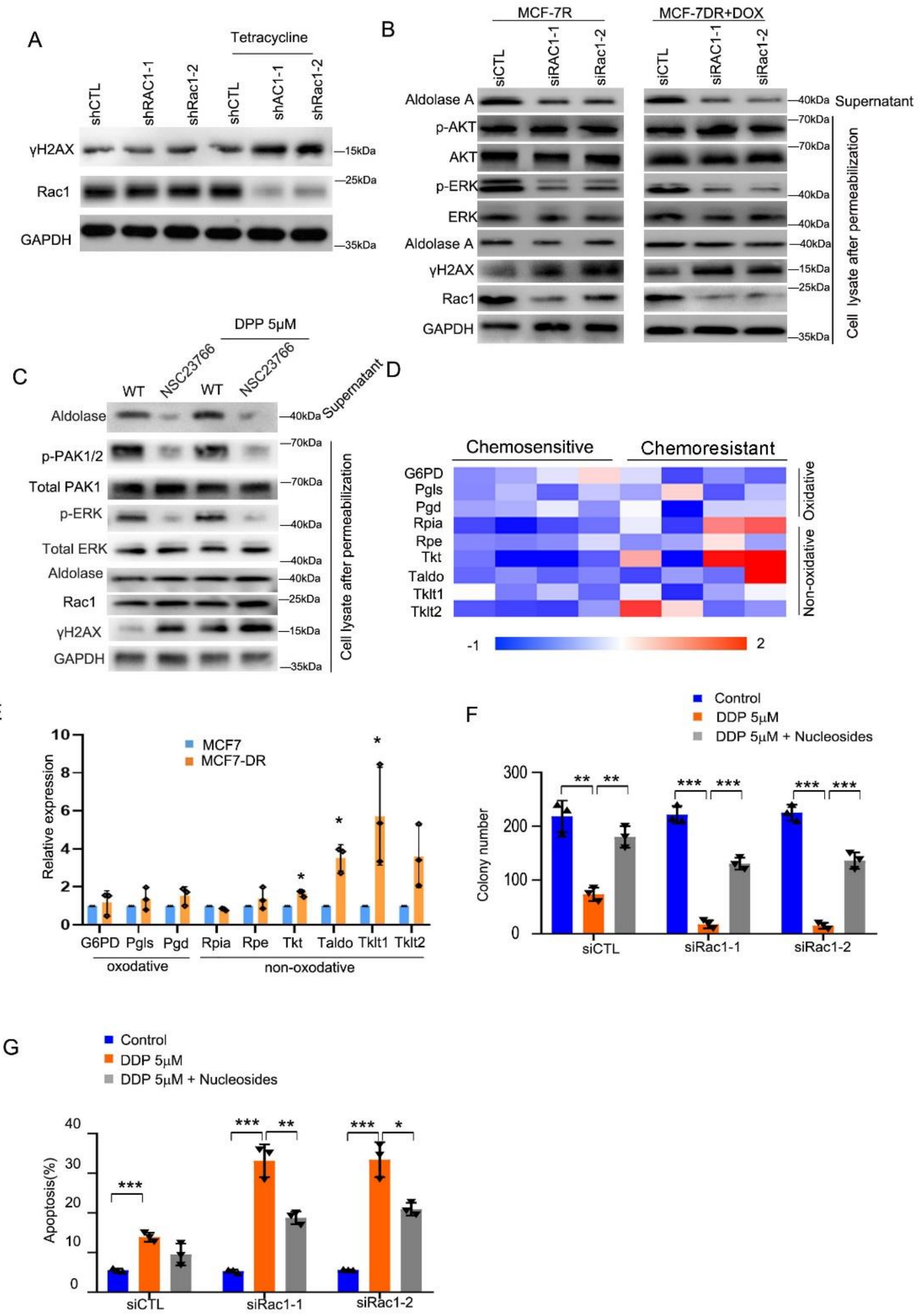

Supplementary figure 4. Rac1 silencing increases DNA damage via suppression of glycolysis and nonoxidative pentose phosphate pathway (PPP) , related to Figure 3, 4 and 5

(A) Immunoblotting of γH2AX in inducible Rac1 knockdown cells. MDA-MB-231 cells were stably transfected with Plko-tet-on-shCTL, Plko-tet-on-shRac1-1 and Plko-tet-on-shRac1-2 plasmids with or without adding 100 ng/ml doxycline for 48h. The result was obtained over three independent experiments.

(B) Rac1 knockdown decreases Aldolase A level and ERK activation in MCF-7DR cells. MCF-7DR were treated with or without doxorubicin (100 μg/ml) 48h after transiently transfected with siCTL, siRac1-1 and siRac1-2. The cells were permeabilized with digitonin (30 μg/ml) for 5 min and then the supernatant and cell lysate were collected separately for immunoblotting.The result was obtained over three independent experiments.

(C) Rac1 inhibition decreases Aldolase A level in supernatant as well as PAK/ERK signaling in MDA-MB-231 cells. MDA-MB-231 were treated with Rac1 inhibitor NCS23766 (50μM) overnight. The cells were permeabilized with digitonin (30 μg/ml) for 5 min and then the supernatant and cell lysate were collected separately for immunoblotting. The result was obtained over three independent experiments.

(D) The expression of PPP related enzymes of tumor samples from patients resistant or sensitive to NAC. The heatmap shows the expression of normalized log2 values.

(E) Real-time PCR for relative mRNA levels of PPP genes in MCF-7DR cells and MCF-7 cells. All mRNA levels were normalized to GAPDH expression. (Tkt p =0.0022, Taldo p=0.0032, Tklt1 p =0.0339, two-sided unpaired t-test). Bar graphs represent the mean ± SD of three independent experiments. *p<0.05.

(F, G) Quantification of colony formation and apoptosis of nucleosides rescuing of Rac1 silencing upon DDP treatment. MDA-MB-231 cells were transiently transfected with siCTL, siRac1-1 and siRac1-2 and cultured in the medium added with nucleosides (A, G, C, U; 100 μM) for 48h and then treated with or without 5 μM DDP for 24h. (Figure F: of siCTL group, control vs DDP, p = 0.0014, DDP vs DDP+Nucleosides p = 0.0014, of si-1 group, control vs DDP, p < 0.0001, DDP vs DDP+Nucleosides p =

0.0001, of si-2 group, control vs DDP, $p < 0.0001$, DDP vs DDP+Nucleosides $p = 0.0002$. Figure G: of siCTL group control vs DDP, $p = 0.0003$, of si-1 group control vs DDP, $p = 0.0003$, DDP vs DDP+Nucleosides $p = 0.0049$, of si-2 group control vs DDP, $p = 0.0004$, DDP vs DDP+Nucleosides $p = 0.0101$, two-sided unpaired t-test).

Bar graphs represent the mean ± SD of three independent experiments. *$p<0.05$, **$p < 0.01$ and ***$p < 0.001$.

Source data are provided as a Source Data file.

Supplementary Figure 5

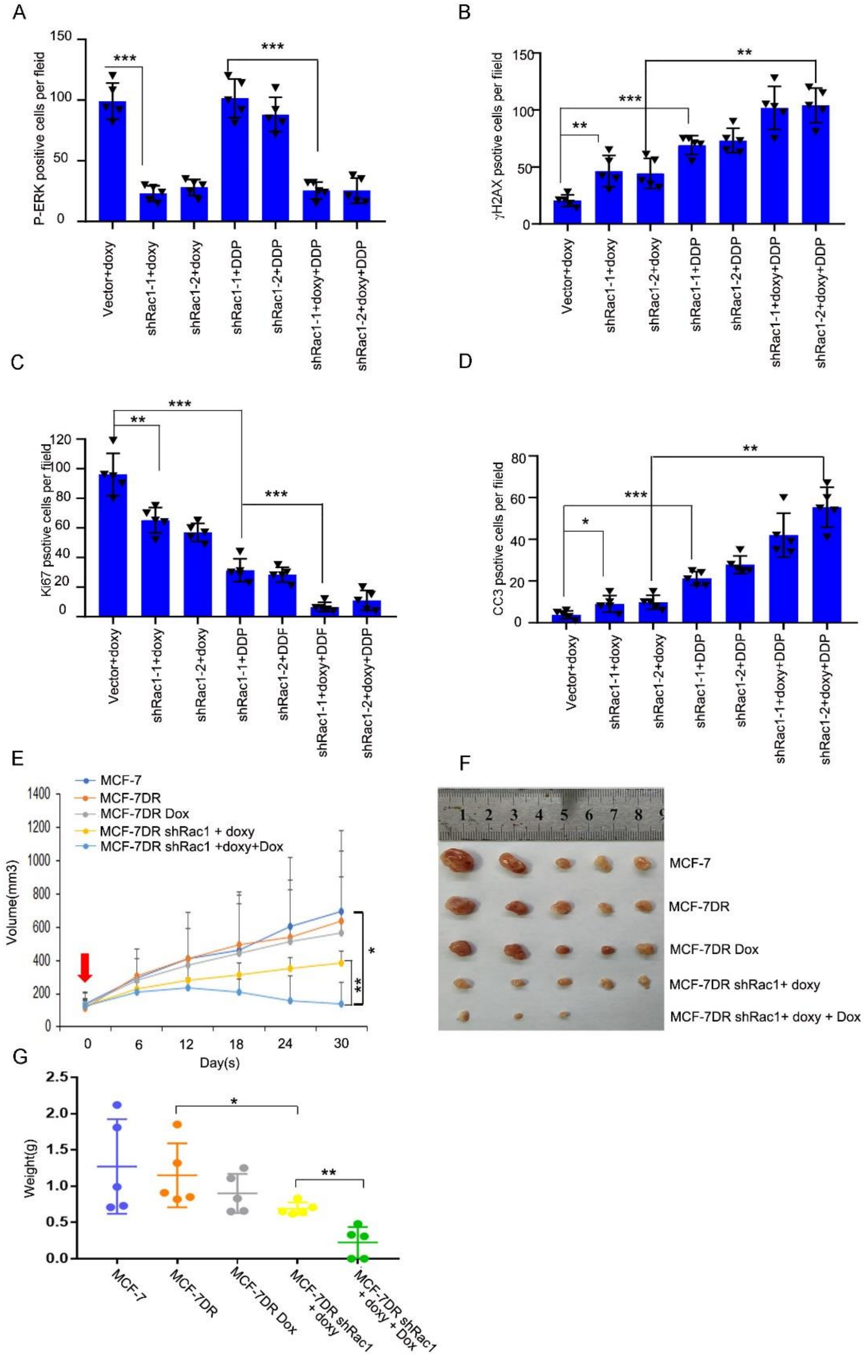

Supplementary figure 5. **Rac1 knockdown increases chemosensitivity of breast tumors, related to Figure 6**

(A-D) Quantitative analysis of DNA damage, cell proliferation and apoptosis in response to shRac1 and DDP treatment. Immunohistochemistry was performed with antibodies against p-ERK (A), γH2AX (B), Ki67 (C) and CC3 (D). These immunostainings in each group (n=5) were analyzed to quantify the positive staining. (A: Vector+ doxy vs sh1+doxy p <0.0001, sh1+DDP vs sh1+DDP+doxy p <0.0001, B: Vector+ doxy vs sh1+doxy p =0.0077, Vector+ doxy vs sh1+DDP p <0.0001, sh2+doxy vs sh2+DDP+doxy p =0.0002, C: Vector+ doxy vs sh1+doxy p =0.0033, Vector+ doxy vs sh1+DDP p <0.0001, sh1+doxy vs sh1+DDP+doxy p =0.0002, D: Vector+ doxy vs sh1+doxy p =0.0307, Vector+ doxy vs sh1+DDP p <0.0001, sh2+doxy vs sh2+DDP+doxy p <0.0001, two-sided unpaired t-test). Error bars show mean ± SD. *p<0.05, **p < 0.01 and ***p < 0.001.

(E) Growth curves of xenografts formed from MCF-7, MCF-7DR and MCF-7DR transfected with inducible Rac1 knockdown constructs. When tumor (n=5 per group) size reached ~150mm^3, mice were injected with doxorubicin intraperitoneally (4 mg/kg weekly), and fed with doxycline (2mg/ml). (MCF-7 vs MCF-7DR sh-1+doxy+DOX p = 0.0487, MCF-7DR sh-1+doxy vs MCF-7DR sh-1+doxy+DOX p = 0.0082, two-way ANOVA + Dunnett's post hoc tests Error bars show mean ± SD),*p<0.05, **p < 0.01 and ***p < 0.001.

(F) Xenografts of MCF-7, MCF-7DR and MCF-7DR cells that harvested 30 days post injection.

(G) Tumor weights of MCF-7, MCF-7DR and MCF-7DR cells harvested 30 days post injection. (MCF-7DR vs MCF-7DR sh-1+doxy p =0.0478, MCF-7DR sh-1+doxy vs MCF-7DR sh-1+doxy+DOX p=0.0034, Tklt1 p =0.0019, two-sided unpaired t-test) Bar graphs represent the mean ± SD of five tumors. *p<0.05, **p < 0.01 and ***p < 0.001. Source data are provided as a Source Data file.

Supplementary Figure 6

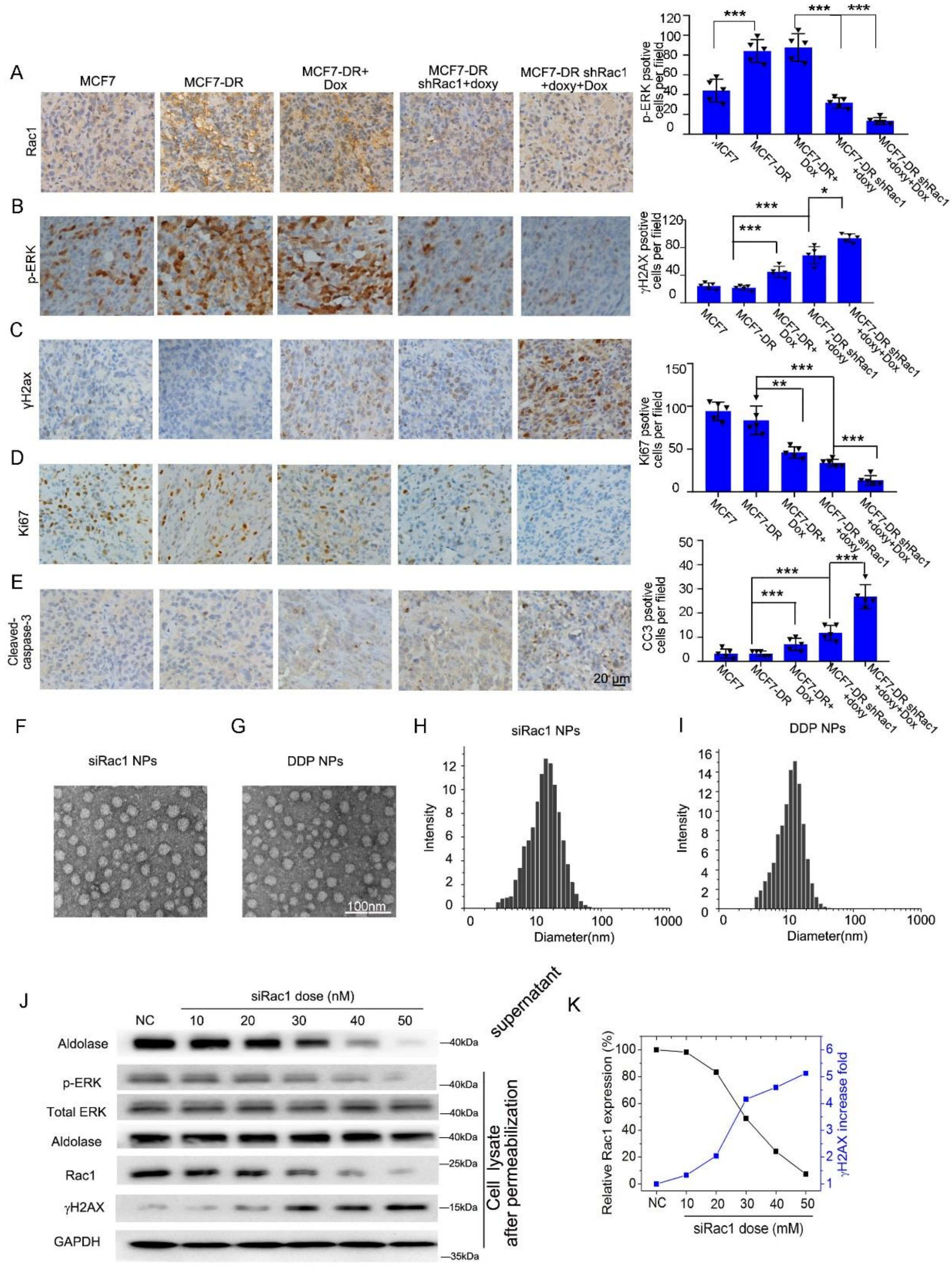

Supplementary figure 6. **Rac1 knockdown decreases the chemoresistance of breast tumors, related to Figure 6 and 7**

(A) Representative immunohistochemical image of Rac1 staining in paraffin-embedded xenograft sections.

(B-E) Representative immunohistochemical image of paraffin-embedded xenograft sections and statistical analysis of immunohistochemical staining on xenograft sections. The staining of p-ERK (B), γH2AX (C), Ki67 (D) and cleaved caspase-3 (E) were counted from five randomly chosen fields. (B: MCF-7 vs MCF-7DR p =0.0006, MCF-7DR+DOX vs MCF-7DR sh1+doxy p <0.0001, MCF-7DR sh1+doxy vs MCF-7DR sh1+doxy+DOX p =0.0002. C: MCF-7DR vs MCF-7DR+DOX p <0.0001, MCF-7DR vs MCF-7DR sh1+doxy p<0.0001, MCF-7DR sh1+doxy vs MCF-7DR sh1+doxy+DOX p =0.0178. D: MCF-7DR vs MCF-7DR+DOX p =0.002, MCF-7DR vs MCF-7DR sh1+doxy p=0.0001, MCF-7DR sh1+doxy vs MCF-7DR sh1+doxy+DOX p<0.0001. E: MCF-7DR vs MCF-7DR+DOX p<0.0001, MCF-7DR vs MCF-7DR sh1+doxy p=0.0006, MCF-7DR sh1+doxy vs MCF-7DR sh1+doxy+DOX p<0.0001, two-sided unpaired t-test).Error bars show mean ± SD of five random fields, Scale bars, 20 µm. *p<0.05, **p < 0.01 and ***p < 0.001.

(F-I) TEM image (F, G) and size distribution (H, I) of the siRac1 NPs or DDP NPs in pH 7.4 PBS solution. The result was obtained over three independent experiments. Scale bars, 100 nm.

(J-K) The expression of Rac1, γH2AX, aldolase A and p-ERK in the mouse breast cancer 4T1 cells treated with the siRac1/DDP NPs at different siRNA doses. The NPs loading with scrambled siRNA and DDP prodrug were used as negative control (NC). The same result was obtained from three independent experiments.

Source data are provided as a Source Data file.

Supplementary Figure7

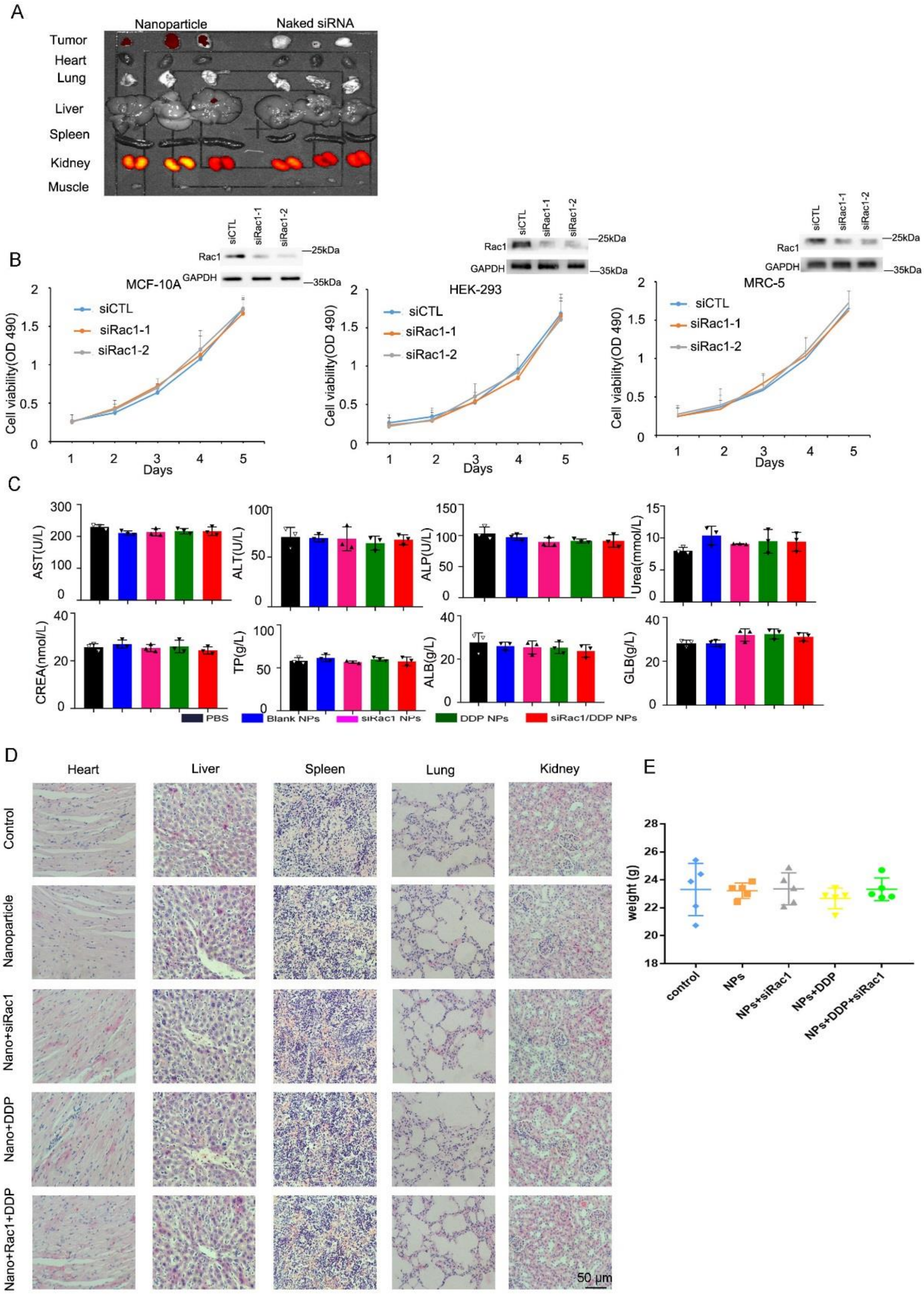

Supplementary figure 7. siRac1/DDP NPs dramatically increases the chemosensitivity of breast tumors but does not induce toxicity *in vivo*, related to Figure 8

(A) Fluorescent images of biodistribution of the Cy5-siRac1 in the tumors and major organs of the MDA-MB-231 xenograft-bearing mouse (n=3) at 24h after injecting naked Cy5-siRNA or Cy5-siRNA-DDP-loaded TCPA2-NPs.

(B) Rac1 silencing shows no effect on the cell proliferation of MCF-10A, HEK-293 and MRC-5, as detected by MTS assay. Bar graphs represent the mean ± SD of experimental triplicates.

(C) Indicated hematological parameters including aspartate aminotransferase, alanine aminotransferase, albumin, alkaline phosphatase, creatinine, and total protein are in the normal range 24 h post indicated treatment. Bar graphs represent the mean ± SD of three independent experiments.

(D) The HE staining of the major organs of the MDA-MB-231 xenograft-bearing mouse (each group n=5). No observable toxicity was found in the tissues from the organs. Scale bars, 50 μm.

(E) Weight of the PDX-bearing mouse post-sacrifice. Bar graphs represent the mean ± SD of five mouse.

Source data are provided as a Source Data file.

Supplementary Figure 8

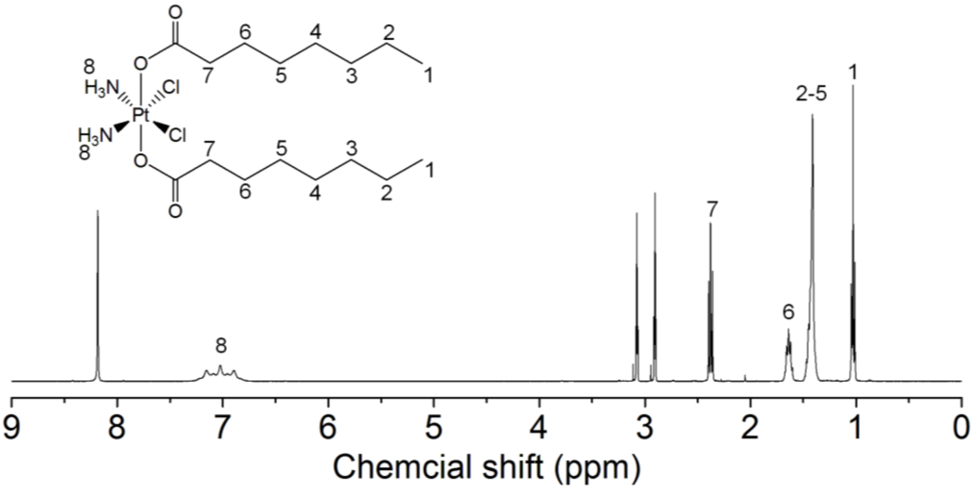

Supplementary Figure 8. HNMR spectrum of the DDP prodrug

The structure of this DDP prodrug was analyzed by proton nuclear magnetic resonance (HNMR) in $CDCl_3$.

Supplementary Table 1 The patients' information of eight breast cancer samples for microarray analysis

Patient	Age	subtype	Ki67(%)	Neoadjuvant chemotherapy regimens	Regimens Cycles	Treatment Evaluation	BRCA1/2 Mutation
NO.1	34	TNBC	30	EC-T	4	CR	NO
NO.2	48	TNBC	5	EC-T	4	CR	NO
NO.3	59	TNBC	15	EP	6	PR	NO
NO.4	45	TNBC	35	ET	5	PR	NO
NO.5	41	TNBC	40	EC-T	4	PD	NO
NO.6	60	TNBC	20	ETC	4	PD	NO
NO.7	56	TNBC	15	EC-T	4	PD	NO
NO.8	53	TNBC	50	CEF	3	PD	NO

Abbreviation:TNBC:Triple negative breast cancer
EC-T:Epirubicin,Cyclophosphamide,Tocetaxel
EP:Etoposide,Cisplatin
ET:Epirubicin,Tocetaxel
ETC: Epirubicin, Docetaxel, Cyclophosphamide
CEF:Cyclophosphamide,Epirubicin,Fluorouracil

Supplementary Table 2. Correlations of Rac1 Expression with Clinicopathological Status in 198 Cases of Patients with Breast cancer

Variable	Rac1		*P* value*
	H-Score≤120(101)	H-Score>120 (97)	
age			
≤35	10	6	0.243
>35	91	91	
Grade			
I+II	54	41	0.076
III	47	56	
T stage			
T1+T2	97	87	0.071
T3+T4	4	10	
N stage			
N0	56	47	0.200
N1+N2+N3	45	50	
M stage			
M0	88	85	0.543
M1	13	12	
TNM stage			
I+II	76	68	0.257
III+IV	25	29	
ER Expression			
Positive	37	40	0.302
Negative	64	57	
PR Expression			
Positive	32	43	0.046
Negative	69	54	
HER2 Expression			
Positive	13	16	0.302
Negative	88	81	
Ki67 Expression			
≤14%	13	12	0.413
>14%	70	78	

Abbreviation: ER: Estrogen Receptor

PR: Progestogen Receptor

HER2: Human Epidermal growth factor Receptor 2

*Examined by chi-square test

Supplementary Table 3 Correlations of Rac1 Expression with Clinicopathological Status in 133 Cases of Breast cancer Patients with Neoadjuvant chemotherapy

Variable	Rac1		*P* value*
	H-Score≤99 (n=73)	H-Score>99 (n=60)	
age			
≤35	11	6	0.273
>35	62	54	
Grade			
I+II	19	7	0.030
III	54	53	
T stage			
T1+T2	62	45	0.112
T3+T4	11	15	
N stage			
N0	18	16	0.473
N1+N2+N3	55	44	
M stage			
M0	66	52	0.342
M1	7	8	
TNM stage			
I+II	40	38	0.207
III+IV	33	22	
ER Expression			
Positive	12	10	0.577
Negative	61	50	
PR Expression			
Positive	9	2	0.056
Negative	64	58	
HER2 Expression			
Positive	16	6	0.052
Negative	57	54	
Ki67 Expression			
≤14%	7	2	0.139
>14%	66	58	
Treatment Response			
CR	28	2	0.028
PR	29	21	
SD	9	21	
PD	7	16	

*Examined by chi-square test

Supplementary Table 4. Multivariate Cox proportional hazard analysis of 198 Cases of Patients with Breast cancer

Variable	x^2	*P* value*	HR	95% CI
Rac1 expression	10.27	0.036	1.577	(1.031-2.413)
age	0.029	0.012	0.382	(0.180-0.811)
Grade	15.48	0.001	2.264	(1.437-3.567)
TNM stage	19.036	0.001	2.858	(1.854-4.406)
Ki67 Expression	3.309	0.029	1.962	(1.072-3.591)

*Examined by Wald test

Supplementary Table 5. Multivariate Cox proportional hazard analysis of 101 Cases of TNBC Patients

Variable	x^2	*P* value*	HR	95% CI
Rac1 expression	10.93	0.013	3.017	(1.263 -7.205)
age	0.05	0.028	0.162	(0.032-0.818)
Grade	9.221	0.057	2.697	0.971-7.496)
TNM stage	2.274	0.010	4.745	(1.067-21.089)
Ki67 Expression	2.374	0.041	3.496	(1.344-9.098)

*Examined by Wald test

Supplementary Table 6. Gene Set Enrichment Analysis of the mRNA expression profiles of the NAC TNBCs

ID	*p* value*	ES	Used.set.size	Term	Size
3010	0	0.78101	84	Ribosome	92
3013	0	0.38778	145	RNA transport	151
3040	0	0.48397	121	Spliceosome	127
4740	0	-0.34801	371	Olfactory transduction	388
4080	3.10862E-15	-0.25748	267	Neuroactive ligand-receptor interaction	272
3008	4.37471E-11	0.41214	73	Ribosome biogenesis in eukaryotes	80
4512	9.55465E-08	-0.32246	82	ECM-receptor interaction	85
4510	1.0336E-07	-0.20924	197	Focal adhesion	200
5414	9.19731E-07	-0.30184	81	Dilated cardiomyopathy	90
3018	2.45535E-06	0.32021	67	RNA degradation	71
4270	4.14564E-06	-0.24579	110	Vascular smooth muscle contraction	116
5410	4.32365E-06	-0.29843	74	Hypertrophic cardiomyopathy (HCM)	83
5322	6.24099E-06	0.22629	126	Systemic lupus erythematosus	136
3030	1.21123E-05	0.42765	33	DNA replication	36
4020	1.40041E-05	-0.18855	171	Calcium signaling pathway	177
3015	1.47919E-05	0.2716	81	mRNA surveillance pathway	83
3420	1.75992E-05	0.38253	40	Nucleotide excision repair	44
190	3.25754E-05	0.21978	116	Oxidative phosphorylation	132
970	4.00677E-05	0.37344	39	Aminoacyl-tRNA biosynthesis	63
4110	6.96541E-05	0.20856	120	Cell cycle	124
4141	0.000145786	0.17667	156	Protein processing in endoplasmic reticulum	165
3430	0.000167607	0.48511	20	Mismatch repair	23
5016	0.000201459	0.16603	171	Huntington's disease	183
4970	0.000317194	-0.23516	80	Salivary secretion	89
5010	0.000320442	0.17082	153	Alzheimer's disease	167
4120	0.000548466	0.1813	127	Ubiquitin mediated proteolysis	135
5012	0.000561008	0.19175	113	Parkinson's disease	130
3020	0.000573076	0.38242	28	RNA polymerase	29
4350	0.00092573	-0.21762	82	TGF-beta signaling pathway	84
3410	0.001033077	0.36197	29	Base excision repair	33
4974	0.00135337	-0.22322	74	Protein digestion and absorption	81
1100	0.0014793	0.062716	1066	Metabolic pathways	1130
5215	0.001651868	0.20208	88	Prostate cancer	89
240	0.001839026	0.1962	92	Pyrimidine metabolism	99
4950	0.001925707	-0.37336	25	Maturity onset diabetes of the young	25
4010	0.002163474	-0.11693	259	MAPK signaling pathway	268
563	0.00241582	0.3828	23	GPI-anchor biosynthesis	25
5412	0.002722978	-0.21665	71	Arrhythmogenic right ventricular cardiomyopathy	74
4744	0.003022244	-0.34747	27	Phototransduction	29
230	0.00369689	0.1459	151	Purine metabolism	162

Top 40 enriched terms are exhibited.

Abbreviation, ID: Gene set ID from KEGG, ES: Enrichment score

**p* value is examined by permutation test.

** Enrichment score is calculated by Kolmogorov–Smirnov test.

Supplementary Table 7. Information of patients whose tumors were used for PDX experiments

Age	Rac1 score	chemotherapy	Treatment cycle	Tumor size (cm)
51	240	TC	4	1.5 X 1
52	210	TP	6	3 X 2.5
33	180	TP	3	3 X 2

T: docetaxel

C: carboplatin

P: cisplatin
