# Supplementary figures and images for: Rac1 activates non-oxidative pentose phosphate pathway to induce chemoresistance of breast cancer
Source: Nat Commun. 2020 Mar 19;11:1456. doi: 10.1038/s41467-020-15308-7 (PMC7081201; doi:10.1038/s41467-020-15308-7)

Fig2

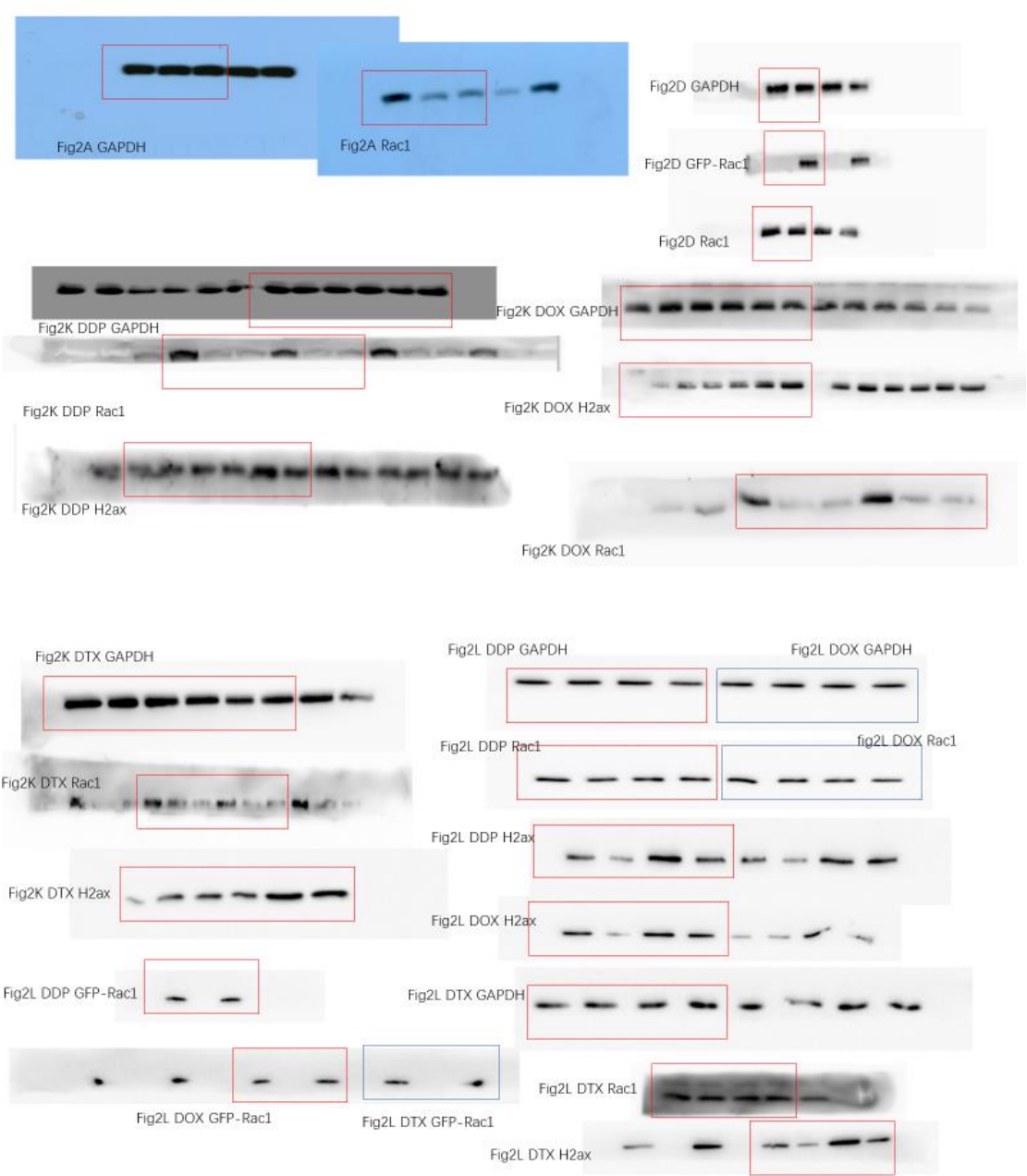

Fig3

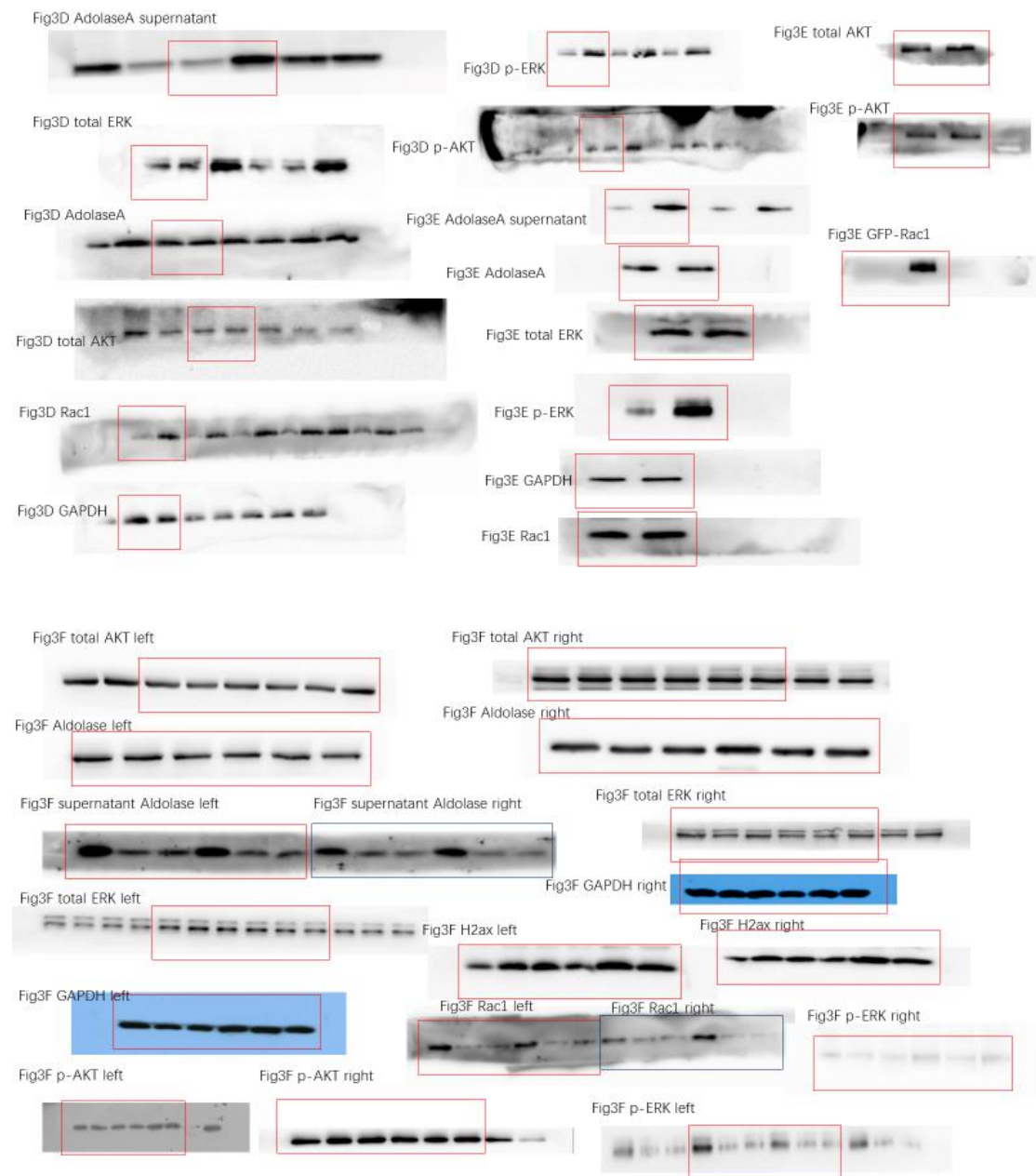

Fig4

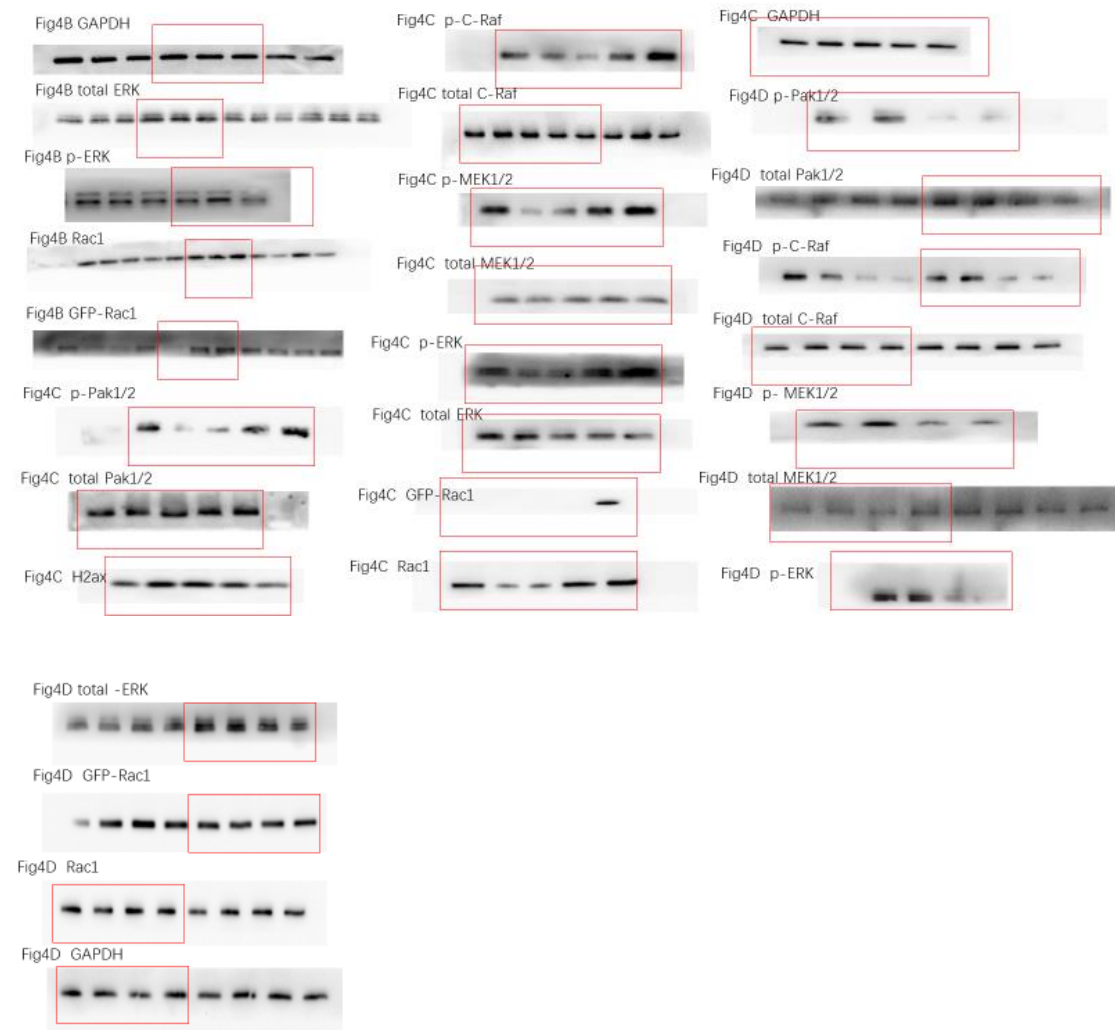

Fig5

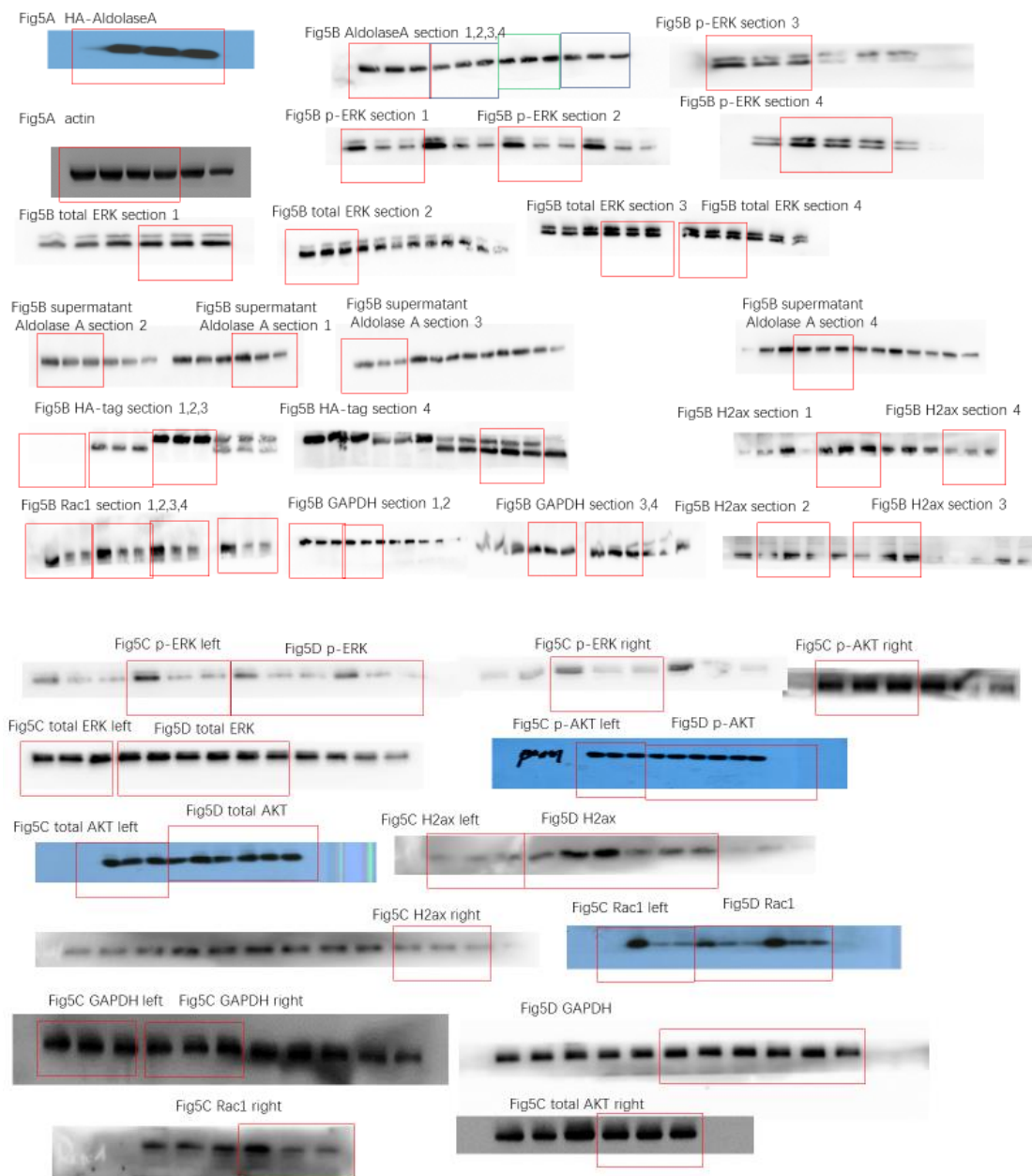

Fig7

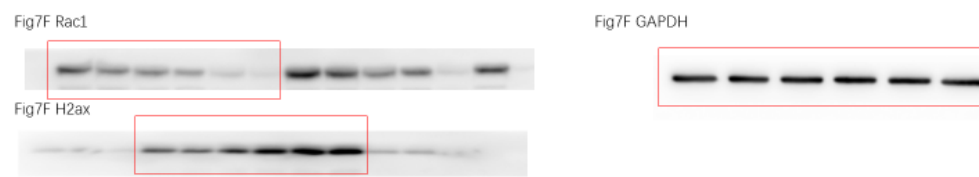

FigS1,2

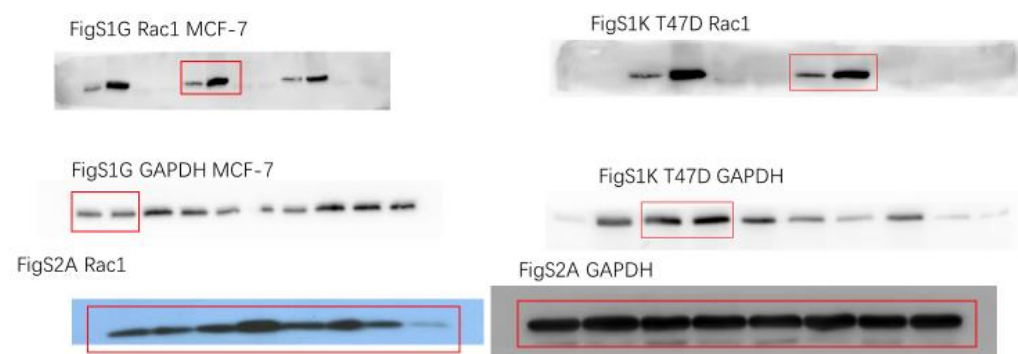

FigS3

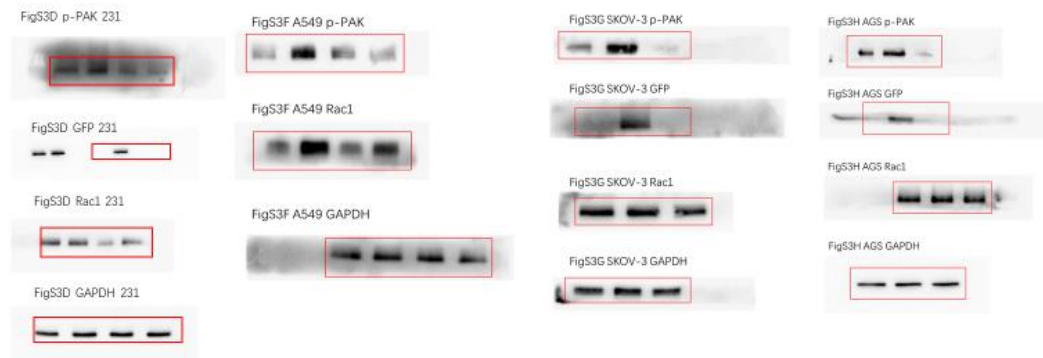

FigS4

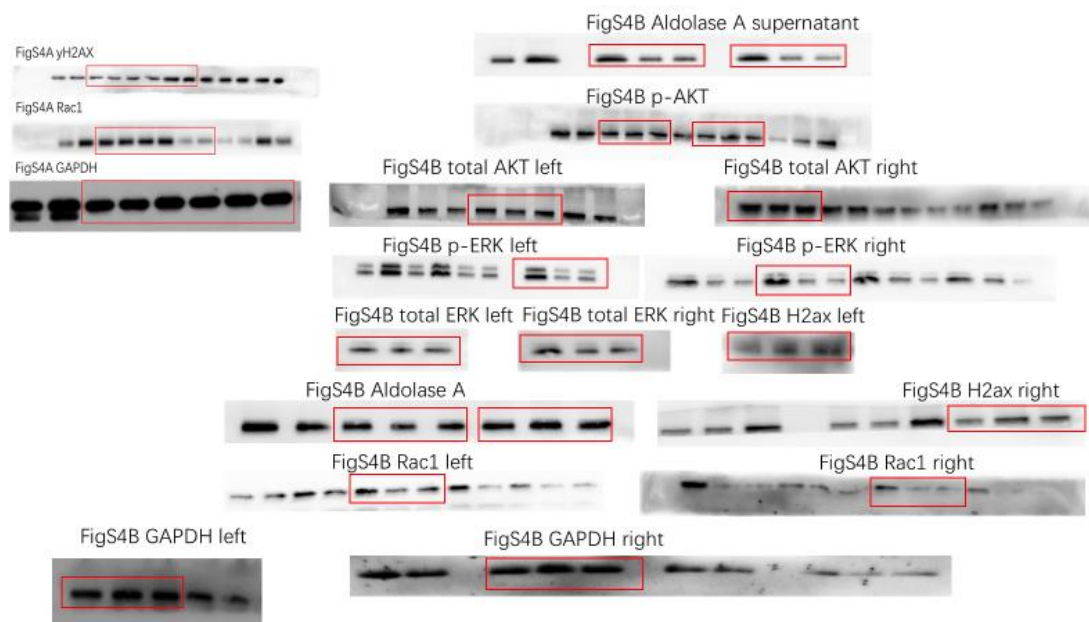

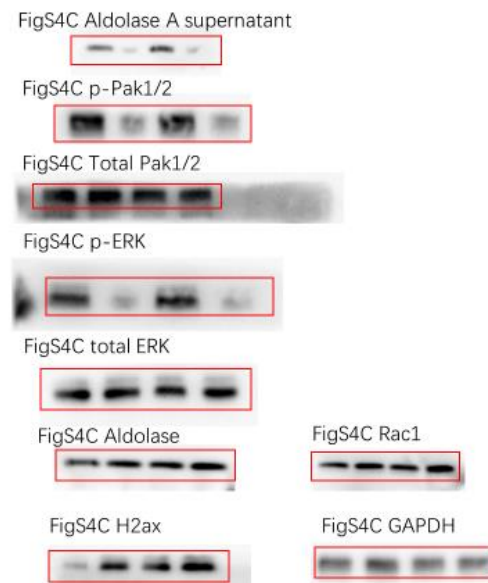

FigS6

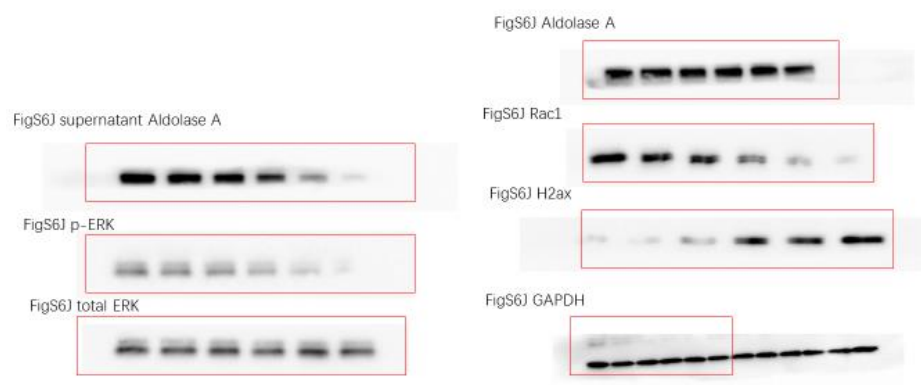

FigS7

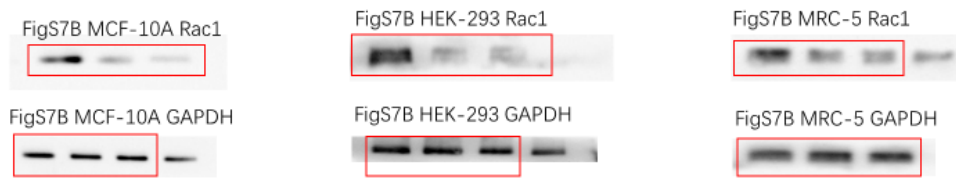

Supplement: Supplementary file 3 — Source Data [file 41467_2020_15308_MOESM3_ESM.pdf]
